# Sorting of Particles Using Inertial Focusing and Laminar Vortex Technology: A Review

**DOI:** 10.3390/mi10090594

**Published:** 2019-09-10

**Authors:** Annalisa Volpe, Caterina Gaudiuso, Antonio Ancona

**Affiliations:** 1 Physics Department, Università degli Studi di Bari ‘Aldo Moro’, Via G. Amendola 173, 70126 Bari, Italy; 2 Institute for Photonics and Nanotechnologies (IFN), National Research Council, Via Amendola 173, 70126 Bari, Italy

**Keywords:** inertial micro-fluidics, inertial focusing, lab on a chip, micro-fluidics, vortex technology

## Abstract

The capability of isolating and sorting specific types of cells is crucial in life science, particularly for the early diagnosis of lethal diseases and monitoring of medical treatments. Among all the micro-fluidics techniques for cell sorting, inertial focusing combined with the laminar vortex technology is a powerful method to isolate cells from flowing samples in an efficient manner. This label-free method does not require any external force to be applied, and allows high throughput and continuous sample separation, thus offering a high filtration efficiency over a wide range of particle sizes. Although rather recent, this technology and its applications are rapidly growing, thanks to the development of new chip designs, the employment of new materials and microfabrication technologies. In this review, a comprehensive overview is provided on the most relevant works which employ inertial focusing and laminar vortex technology to sort particles. After briefly summarizing the other cells sorting techniques, highlighting their limitations, the physical mechanisms involved in particle trapping and sorting are described. Then, the materials and microfabrication methods used to implement this technology on miniaturized devices are illustrated. The most relevant evolution steps in the chips design are discussed, and their performances critically analyzed to suggest future developments of this technology.

## 1. Introduction

Many research studies in the biomedical field need to analyze individually specific cell types or micro-particles, contained in heterogeneous samples. For this purpose, the target components have to be isolated and sorted from the original mixture, in order to allow cell analysis. Stem cells studies, tissue and organs regeneration [1,2,3,4], lethal disease diagnosis and therapy [5,6] and drug screening [7] are made possible, thanks to the development of isolation, sorting and separation techniques for specific target cells or micro-particles. As an example, isolating rare circulation tumor cells (CTCs) from blood allows early an diagnosis and prognosis of cancer disease and its progression [5,8,9,10,11].

Conventional techniques like fluorescence-activated cell sorting (FACS), magnetically-activated cell sorting (MACS) and centrifugation methods have been proven successful to separate and extract the targeted cells. Nevertheless, they are affected by some technical limits, since they need labeled cells, long processing times, high operating costs and skilled personnel, despite having a low efficiency [12].

Significant advantages have been introduced by micro-fluidics and lab-on-a-chip (LoC) technology, which promotes novel techniques for cells sorting in a downsized scale. In fact, thanks to their reduced dimensions, LoCs ensure a good portability, paving the way for building a miniaturized platform for medical analysis, thus making this technology suitable for rapid diagnostics and therapy.

Active and passive methods have been implemented in micro-fluidic devices for cell sorting purposes: In the first case, external field forces are applied to the fluid sample to sort cells. On the other hand, passive methods do not employ external forces, but do rely upon the inherent characteristics of the cells, such as shape, dimensions, deformability [13,14,15].

Among the active methods, acoustophoresis (acoustic levitation) is based upon applying an acoustic force, by means of continuous ultrasound waves [16]. Here, particles suspended in a liquid are exposed to an acoustic standing wave, which causes them to move in the sound field. The magnitude of the exerted force depends on the features of particles, such as size, density and deformation. Therefore, cells separation can be obtained according to these characteristics [17,18,19,20]. However, care should be taken due to the large amount of ultrasound, causing temperature increase which can damage the cells, thus affecting their viability. Nevertheless, CTCs separation with a recovery rate of 83% at a throughput of 10^3^ cells has been reported in [21]. Dielectrophoresis (DEP) uses an electric field as an external excitation source and relies on the dielectric characteristics of most of the biologicals cells in suspension. The magnitude and the direction of the exerted force is determined by the polarizability of the cells, besides the applied electric field. Therefore, a continuous electric field affects the cells’ motion, and is capable of moving particles in different equilibrium positions. High purity (84%) and efficiency (92%) have been reported in [22] the case of stem cells. Magnetophoresis, instead, is based on the use of an external magnetic field to separate particles. In this case, magnetic beads attached to the target cells can be moved under the effect of a strong magnetic field, usually generated by a permanent magnet or electromagnets. This method has been reported for example for *Escherichia coli* (*E. coli*) separation from bovine blood [23], and for removing blood cells infected by malaria [24]. However, label-free magnetic methods have been also reported, for separating red blood cells (RCBs) and white blood cells (WBCs) [25,26], exploiting their native magnetic properties.

Optical methods have also been demonstrated very successful in cell sorting. Here, the optical force depends upon the refractive index and the size of the particles, and can be used to separate cells according to these features [27]. In particular, its magnitude decreases as the refractive index of the particle compared to the host fluid and its volume reduces. Therefore, within the photon description of light, each photon travels according to a geometrical optics ray description, and transfers momentum to the particles in the sample fluid by mean of collisions (elastic or inelastic) [28]. One of the most common optical sorting methods is the optical tweezer, where only one highly focused laser beam is used to exert a force on particles [29]. The possibility of manipulating particles of different dimensions relies on the capability of focusing a Gaussian beam on a very small area, i.e., the beam waist, where a strong electric field gradient is generated which attracts particles and makes them change their motion. Despite the Gaussian beams are the most used for such an application, being the fundamental selectable laser mode, also other transverse modes have been used, e.g., the doughnut beam [30].

Thanks to its simplicity, microfiltration sets as the most known passive method for cells sorting. Using membranes [31,32], pillars [33,34] and weirs [35], this microfiltration allows cell separation depending on their size. Among the advantages of these methods, the simple structures, the high separation efficiency and the precise control achievable with this kind of device, can be highlighted, despite suffering from clogging, which strongly affects their durability.

Hydrodynamic forces and channel shape are the key elements of the deterministic lateral displacement methods (DLD) [36], capable of separating cells depending on their sizes. This method uses the laminar flow hypothesis for the fluid sample motion. Therefore, the target particles suspended in the fluid move according to parallel streamlines, which define the parallel adjacent layers of the fluid. Thus, by placing on their trajectories arrays of obstacles in different directions with respect to the sample flow, the streamlines are bent, and the particles separated. A critical diameter determined by the size of the obstacles, their mutual distance and the distance at which they are separated after each row, defines the performances of the device. In fact, particles smaller than this critical diameter can be separated from bigger ones.

In this case, diffusion can limit the efficiency of these devices, leading particles to move laterally with respect to the streamlines, towards other rows, thus causing a mix between particles and the reduction of selectivity. However, a recovery of 99% and a cell viability higher than 87% [37] have been reported in the case of CTCs clusters isolation.

Recently, the possibility to exploit inertial effects for cell sorting has also been investigated, and several inertial effects are beginning to find a utilization in micro-fluidic systems. The predominantly used effects for inertial sorting are: (i) Inertial migration of particles in a straight channel and (ii) secondary flows in curved channels. As we will better describe in Section 2.1, inertial focusing in straight channels consists of particles’ migration across streamlines, due to the onset of inertial forces whose intensity depends upon the particles size [14,38,39,40,41,42,43]. This phenomenon has been extensively studied for Reynold numbers between 1 and 1000 [44,45,46]. In this regime, the inertial effects become predominant on the viscous one, but a laminar flow is always possible.

Inertial focusing in a straight micro-channel has found various applications thanks to its ease of fabrication and use. High aspect ratio straight rectangular channels have been used for example by Hur et al. [47] for separating the murine adrenal cortical progenitor from the adrenal gland digest. In particular, they have shown that the inertial focusing depends on the particles’ dimensions, finding that bigger cells were collected close to the channel center and separated from the smaller ones flowing in positions closer to the channel walls. Di Carlo et al. [48] have reported about the counting and differentiation process of red and white blood cells, with a device consisting of 256 high aspect ratio straight rectangular channels. A novel channel geometry has been introduced by Mach et al. to separate pathogen bacteria cells from blood [49]. They proposed to gradually expand the channel section. In particular, their device consisted of 40 single straight channels, each of them having three segments with different cross-sections. The parallel channels allowed a high throughput sorting process, with a >80% removal of pathogenic bacteria from blood after two passes of the single channel system. Zhou et al. [50] have in turn proposed a device designed on a two-stages inertial migration principle [51]. First, the sample fluid flows through a channel characterized by a high aspect ratio. Here, the particles are focused near the side walls, at half of the channel height. The second channel has a lower aspect ratio, thus focusing the particles at the center. The size-based differentiation between particles is achieved by the higher speed of the bigger particles, which allows them to relocate very quickly, keeping the smaller ones almost unaltered. Rectangular channels with innovative design have also been successfully exploited for other types of particles besides the cells. For example, Li et al. [14] have realized a device for the inertial focusing of a single-celled alga, *Euglena gracilis*, by using a straight channel followed by a gradually expanding chamber and five outlets.

Though particle migration in straight channels has been deeply studied, devices based on this principle are characterized by long channels, which leads to high resistance to the flow and limits the possibilities of miniaturization. That is why devices based on the secondary flow effect, known as “Dean flow”, and originated by channel curvature changes, have been introduced.

The secondary flow effect strongly depends on the particle size; therefore, when two different particles are injected into a curved channel, they can be separated based on their different equilibrium positions. On this basis, Di Carlo et al. have demonstrated the focusing and sorting of cells under laminar flow using a serpentine pattern [12].

Kuntaegowdanahalli et al. [52] demonstrated, for the first time, the size-dependent focusing of particles at distinct equilibrium positions across the micro-channel cross-section in a spiral LoC. The device enabled them to separate neuroblastoma and glioma cells with 80% efficiency and high relative viability (>90%). The isolation and retrieval of circulating tumor cells using centrifugal forces in a spiral channel has been demonstrated by Hou et al. [53]. They obtained 85% cancer cell recovery from blood using an approach named Dean flow fractionation. Similarly, the study of Nivedita et al. [54] reports on an optimized Archimedean spiral device which, in few centimeter squares, reached a separation efficiency of about 95%, and high throughput with rates up to 1 × 10^6^ cells per minute.

Warkiani et al. [55] successfully demonstrated CTC isolation and detection in blood samples of patients with a metastatic tumor, achieving ≥85% recovery of spiked cells across multiple cancer cell lines, and 99.99% depletion of white blood cells in whole blood. Further studies have been conducted by Guan [56] et al. and Warkiani et al. [57] to enhance the sorting performance of the spiral device, optimizing the channel cross section.

A comprehensive study on spiral inertial micro-fluidics for cell separation and biomedical applications can be found in [58].

Despite all the existing works on inertial particles sorting, the capability of significantly concentrating the target population into a smaller volume after operation is still unsatisfactory [44,59]. This issue has been addressed by combining inertial focusing in a straight channel, together with microscale laminar vortices, as first theoretically proposed in [60,61]. The combination of inertial focusing and this vortex trapping technique presents several advantages with respect to other sorting methods in performance, fabrication and operation. It does not need any filtering systems [62], obstacle structure or mechanical or electrical parts. Furthermore, the micro-fluidic system can be easily operated by only one syringe pump without the use of sheath flow, and aims to be a high throughput method by appropriately designing the devices.

Considering the growing interest in sorting methods based on inertial focusing and the continuous improvements implemented by groups all over the world, a review summarizing the evolution, achievements and indicating the perspectives of this method would be beneficial for the scientific community working in this field. Different reviews con be found in literature dealing with inertial effect in micro-fluidics. One of the most cited and complete one is by Di Carlo [41], which however does not include all the most recent results. Also devoted to inertial micro-fluidics is the review of Zhang et al. [40], where the perspectives of employing fluid inertia in micro-fluidics for particle manipulation are also discussed. Here, the inertial focusing with vortex technology is only partially treated.

The reviews of Sajeesh et al. [63] and Shields et al. [15] are specifically focused on micro-fluidics for particle sorting. Both of them provide to the reader a very comprehensive panorama of the developments in LoC cell sorting, reserving one section to the passive and label-free methods, but without mentioning the vortex technology, which has been only recently developed.

More recent is the Dalili et al. work [64]. Here, inertial-based devices specifically devoted to the biomedical application are treated. Unfortunately, most of the latest results exploiting vortex technology are missing, and the evolution of this method is not deeply discussed.

This review article aims to give a comprehensive overview on the development and diversity of this technology, which is still missing in literature, and in addition, to provide perspectives for future directions. We focus on the inertial sorting combined with microscale laminar vortices technology, by first explaining the physical mechanisms underlying inertial focusing. Successively, the particle trapping obtainable by laminar vortex is described, by highlighting the capability of such an approach for cell enrichment. Materials and methods usually employed for the LoC fabrication and testing are then illustrated.

What follows is a description and critical analysis of the most relevant evolution steps in the chips design. In this section, only the works strictly inherent to inertial focusing in combination with laminar vortices are treated, in order to provide to the reader a linear and clear picture of this technique, serving as a basis for future improvements.

## 2. Inertial Focusing and Laminar Vortex Technology

### 2.1. Working Principle

**Figure 1:**
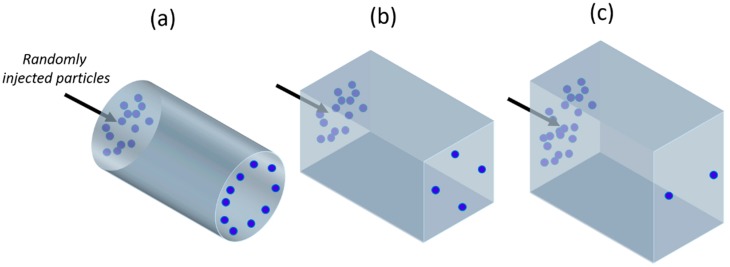
A schematic of inertial particle focusing in a (**a**) tubular, (**b**) square, and (**c**) rectangular channel.

Segrè and Silberberg were the first in 1961 to observe that macroscopic, neutrally-buoyant, spherical particles, diluted in a liquid passing a laminar flow through a straight channel, collect in a thin, annular region at a position 0.6 times the radius of the pipe [65,66]. They experimented that the effect was proportional to the mean velocity of the flow *U_m_*, and to the fourth power of the ratio of particle radius *r* to the tube radius *R*, and independent of the overall particle concentration at least up to the largest volume fraction 0.4% they used. Furthermore, the results were symmetrical around the axis (Figure 1a) of the tube, and were independent of the shape of the mouth of the tube.

Further works have demonstrated the same particles’ focusing effect also in straight, non-circular cross-section micro-channels, e.g., square (Figure 1b) [67], rectangular (Figure 1c) [68], triangular [69], non-conventional shape [70] and gradually changing geometrical structures [38], providing also numerical methods for the fast prediction of the focusing patterns of particles along the channels’ cross section [71,72]. Several experimental results on curved [73], serpentine [74] and spiral [55] micro-channels are also reported.

The ease of fabrication and the reduced number of equilibrium positions (only two located near the widest faces of the channel) have made the straight micro-fluidic channels with rectangular cross sections one of the mot studied geometries for inertial focusing [75].

Regardless of the geometry, different studies have tried to explain the origin of the focusing effect [76,77], bringing it back to the fluid inertia and drawing the particles as spheres. Two types of forces exert on neutrally buoyant particles randomly suspended in a laminar fluid flowing through a micro-channel, i.e., viscous drag forces and inertial lift forces [76]. The firsts, due to the viscous nature of the carried fluid, allow the particles to entrain along the flow streamlines. The parabolic nature of the laminar velocity profile in Poiseuille flow produces a shear-induced inertial lift force (*F_LS_*) [59], pushing the particles away from the micro-channel center, or rather leading them towards the walls. Here, *f_L_*, *a*, and *W_c_* are the dimensionless lift coefficient, the particle diameter and the channel width (i.e., shorter face of the rectangular channel), respectively.

(1) $$F_{LS}=\frac{f_{L}\rho U_{m}^{2}a^{3}}{W_{c}}$$

On the other hand, particles experience a wall-effect-induced lift force (*F_LW_*) [59]

(2) $$F_{LW}=\frac{f_{L}\rho U_{m}^{2}a^{6}}{W_{c}},$$

which becomes more prominent as particles migrate closer to the channels’ walls in two different ways. First, the wall generates an extra drag force making the particles lag behind the fluid. The bounded sphere undergoes a slower relative fluid velocity on the wall side than on the centerline side, thus causing an increased pressure upon the wall side, which pushes the sphere away. Second, the presence of the adjacent wall alters the streamlines around the sphere causing a dissymmetry of the wake vorticity distribution generated around its surface. This phenomenon induces a higher pressure on the wall side, repulsing the sphere away from the wall [78].

**Figure 2:**
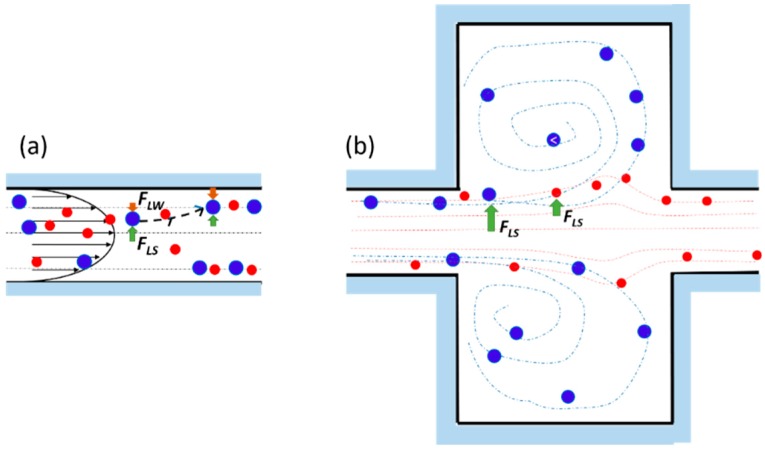
(**a**) The particles randomly flowing in a straight channel undergo two opposite lift forces: The wall effect lift force *F_LW_* and the shear-gradient lift force *F_LS_*, resulting in lateral equilibrium positions depending on the channel section. (**b**) At the entrance of the reservoir, the particles experience *F_LS_*, which increases with their size: The larger blue particles are pushed toward the vortex center and trapped, meanwhile the smaller red ones flow in the main stream. Adapted from [83].

The equilibrium position of the lateral migration is then achieved by the balance of the two opposite lift forces *F_LS_* and *F_LW_* acting bi-directionally on a sphere (Figure 2a) [44,75].

The effect of the inertial forces increases proportionally to the flow velocity and to the particle size relative to the channel dimension. The particles’ Reynolds number *Re_p_* gives an estimation of this fluid dynamic phenomenon. This number represents the particle motion in a channel flow, and can be expressed as [75].

(3) $$Re_{p}=Re_{c}\frac{a^{2}}{D_{h}^{2}}=\frac{U_{m}a^{2}}{vD_{h}},$$

where *Re_c_* is the channel Reynolds number, defined as the ratio of inertial force (density *x* velocity) to viscous force (viscosity/length-scale) ($Re_{c}=U_{m}D_{h}/\nu$); *D_h_* is the hydraulic diameter of the channel, *U_m_* is the mean flow velocity and *ν* is the kinematic viscosity of the fluid, while *a* is the particle diameter. The particle Reynolds number is additionally dependent on the particle size. The inertial lift forces become dominant to drive the particles in the equilibrium position when *R_ep_* > 1, conversely when *R_ep_* << 1 the drag force promotes the longitudinal particle migration.

In case of a channel expansion, the wall effect lift force becomes negligible due to the longer distance of the particles from the sidewall. As a consequence, the particles undergo a non-equilibrium situation, being pushed by the shear lift force in the expansion volume (b). Since the shear-gradient lift force scales with *a*^3^, larger particles experience a stronger lateral lift force than the smaller ones. Furthermore, assuming that *F_LS_* is balanced by the viscous drag force ($F_{D}=3\pi \mu aU_{L}$), the lateral migration velocity of particles scales with particles size as $U_{L}\propto U_{m}^{2}a^{2},$ consequently the larger particles migrate across the streamlines faster than the smaller ones.

When the Reynolds number is sufficiently high, multiple microscale laminar vortices (i.e., Moffatt’s corner eddy flow [79]) are created in the chamber. In such situations, particles with different size are subjected to different lateral migration, which drives them across streamlines crossing the boundary between the main flow and the vortex (separatrix), where they remain isolated, orbiting in it. Therefore, size-selective trapping is achieved. Indeed, particles do not migrate at a sufficient rate to pass the separatrix if their size is below a cutoff *a_c_*, and thus they remain in focused streams, flowing out of the device. Theoretical analyses have shown that the lateral equilibrium position of focused particles shifts toward the wall as *Re_c_* increases. This effect brings the focused streams of flowing particles closer to the boundary streamline at the reservoir and enhances the capturing efficiency proportionally to their size [77].

### 2.2. Fabrication and Testing Methods 

The core of the sorting device based on inertial focusing and laminar vortex technology usually consists of a rectangular cross-section micro-channel with an expansion (micro-chamber). Different channel and chamber dimensions have been investigated, but usually a high-aspect ratio channel of height *H* around 70–100 μm and width *W_c_* of 40–50 μm are used. Indeed, high aspect ratio channels restrict the particles’ equilibrium positions to two, in order to achieve an efficient cell sorting.

The chamber has the same height of the channel and width *W_R_* of about 400–600 μm. The micrometric dimensions of the channels require techniques capable of achieving micrometric resolution and a high quality of the edge.

The most used technique to rapid prototyping lab on a chip is soft lithography [80], due to its high resolution and simplicity. Accordingly, the devices are made of polydimethylsiloxane (PDMS), an elastomeric polymer often used in LoC fabrication due to its optical transparency, thermal stability, low cost, biocompatibility, gas permeability and its ease of fabrication. The conventional procedure to fabricate micro-fluidic patterns using a standard soft-lithography technique requires the prototyping of a master template, PDMS replica molding and plasma oxidation [81,82]. The master template is made using a mask in contact photolithography.

In order to avoid the expensive and time-consuming production of masks and the necessity of a clean room, femtosecond-laser micromachining technology (FLM) for the rapid prototyping of inertial sorting LoCs has been developed [83]. Thanks to the short timescale which characterizes the energy deposition during femtosecond-laser irradiation, material is removed with a negligible heat-affected zone, thus allowing micro-milled features with high precision and resolution [84] to be created. Moreover, being a non-clean room process, FLM provides an economical and flexible way to fabricate micro-fluidic patterns by simply varying the laser parameters. Moving the beam, designs are directly transferred from a computer file to the device, in a one-step fabrication protocol.

The fabricated and sealed micro-fluidic chips can be firstly tested by injecting dry polystyrene microspheres (density ~1.05 g/cm^3^) into the micro-fluidic channels using a syringe pump at a specific flow rate, usually from a few μL/min to a few mL/min, in order to work in a laminar regime where the net inertial forces are strong enough to affect the particle motion [44,45,46]. The microspheres have diameters ranging from 1 to 15 μm, depending on the target application [81], and are suspended in an aqueous solution. The beads’ density is close to the density of the liquid in which they are suspended in order to consider them neutrally buoyant. In the case of devices without lateral exit channels [62,85], the beads captured in the vortices are removed from the device (releasing step) by a final flush at a lower flow rate.

As soon as the device is validated with synthetic beads, it can be tested for the specific application with cells. For example, Hur et al. successfully validated such inertial devices separating breast cancer cells and cervical cancer cells spiked in blood [62]. Sollier et al. [85] extracted and enumerated CTCs from the blood of patients with breast and lung cancer.

### 2.3. Evolution of the Devices Design 

**Figure 3:**
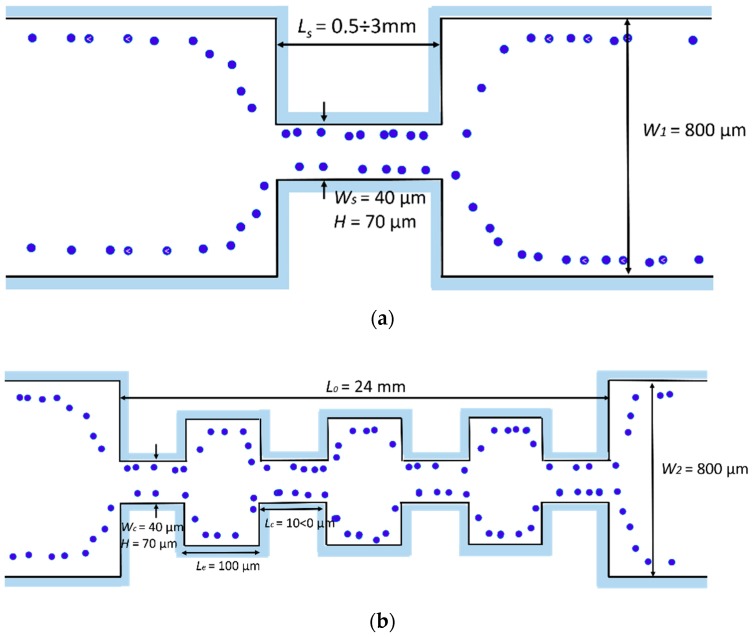
Design of the micro-channels used by Park et al. [81]. (**a**) Straight square channel. For the experiments, the micro-channel length was varied between 0.5 and 3 mm. (**b**) Multi orifice channel. All the structures are 70 µm high (*H*). Adapted from [81].

The inertial lift forces combined with turbulent secondary flows generated in a topographically-patterned micro-channel was exploited in a new and simple way by Park et al. [81]. Their novel micro-fluidic method for focusing micro-particles exploited only the hydrodynamic forces exerted on suspended micro-particles as they passed through a series of contracting and expanding micro-channels. The influence of the micro-channel geometry was investigated on the particle focusing performance. In particular, two geometries were developed: (i) A straight micro-channel (Figure 3a), (ii) a multi-orifice micro-channel (Figure 3b).

**Figure 4:**
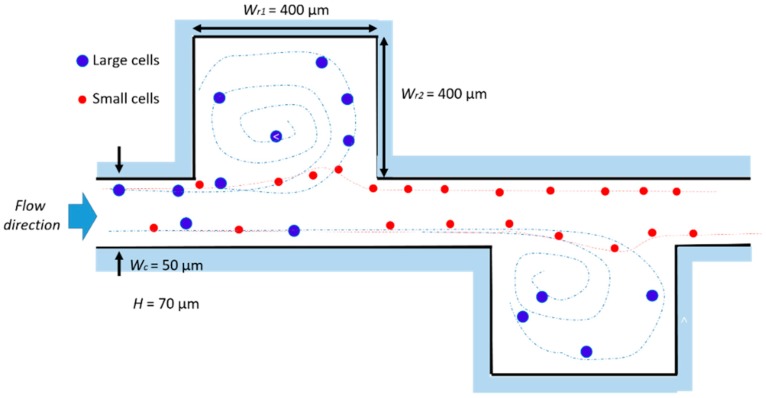
Design and working principle of the device of Hur et al. [56]. The larger cells remain trapped in the lateral chamber, while the smaller ones flow within the main channel, due to the dependence of the lift force on the particles’ dimension. *Wr*_1_ and *Wr*_2_ indicate the chamber dimensions, and *Wc* is the chamber width. All of the structures are 70 µm high (*H*). The dashed paths indicate the fluid streamlines followed by the large (blue) and small (red) particles. Adapted from [62].

The authors observed how these innovative geometries generated secondary flows, i.e., vortices, with a low Reynolds number at the corners of the expansion chambers. This vortex formation depended on several factors, including flow velocity, surface roughness and the cross sectional area of the chamber. Computation fluid dynamic simulations helped to study vortex shape as a function of these parameters. The experimental tests, in agreement with the simulations, showed analogous vortex geometry as a function of the channel Reynolds number. The vortex zone increased with the *Re_c_*, covering almost all of the cavity area when *Re_c_* is over 78. The particle distribution as a function of the *R_ep_*, i.e., of the flow rate, has been extensively studied. For a *Re_p_* of 0.78–2.33, two distinguished distributions near the chamber expansions were observed, which disappeared as the *Re_p_* increased due to the high-speed vortex flow generated in the expansion chamber. This phenomenon was ascribed to the vortex developed in the expansion at a high flow rate, generating a pressure in the main stream, which can substitute the wall-effect lift force. Since all of the mentioned tests have been carried out with a fixed particles’ size, the description of the relationship between the particle Reynolds number and the focusing process was not possible from the experimental data. However, the authors suggested that continuous, non-intrusive, and high throughput techniques usable with real bio-samples can be built by exploiting the hydrodynamic focusing method in multi-orifice channels. Hur at al. presented a passive, continuous micro-fluidic device that isolated larger cancer cells from the smaller blood cells [62]. The core of the device was essentially a single straight high-aspect ratio channel (*W_c_* = 50 μm, *H* = 70 μm and *L* = 4.5 cm), consisting of one inlet with coarse filters, two reservoirs/micro-chambers (*Wr*_1_ = *Wr*_2_ = 400 μm), and one outlet (Figure 4). The high-aspect ratio straight channel was implemented so that flowing particle/cells were inertially focused to two distinct lateral focusing positions closer to the channel walls. To increase the device efficiency, the tests were conducted on a device with eight parallel channels, each with ten trapping reservoirs.

As found by [81], the vortex geometry highly depended upon the flow rate, i.e., the vortices gradually increased with an increasing flow rate, up to *Re_c_* = 215, where the vortices occupied the entire reservoir. In addition, the authors empirically found that particles/cells with a diameter greater than a critical value *a_c_* experience a shear-gradient lift force *F_LS_*, which was strong enough to direct those particles into the reservoir, while the smaller particles remained in the main stream. The critical diameter depended on the *Re_c_*, i.e., decreased when *Re_c_* increased, and it is even greater for living cells with respect to synthetic beads. This outcome suggests that deformation-induced lift forces co-act in superposition with inertial lift forces, thus leading to modified lateral equilibrium positions, which result, dependent upon particle deformability. Consequently, deformability-induced target cell enrichment can be aimed based on the different lateral equilibrium positions among cell types and addressing the entrained target cells to distinct designated outlets. The maximum number of particles that each reservoir could trap and sustain was found to be 25, achieving an enrichment of cancer cells to a factor of up to 7.1, with a capturing efficiency of 23%. The authors suggested that these values can be further enhanced by simply parallelizing more channels or optimizing the reservoir geometry.

**Figure 5:**
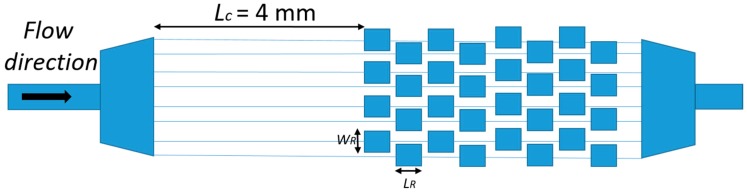
Vortex chip design of Sollier et al. [83]. The chip consists of eight channels (40 um wide, 80–85 um high) in parallel, with eight reservoirs (*W_R_* = 480 um, *L_R_* = 720 um) for each of them. The input channels of length *L_C_* allow the focus of the randomly injected particles. Adapted from [85].

Sollier et al. [85] further improved this design using a vortex chip composed of 64 reservoirs, with eight paths in parallel and eight reservoirs per path (Figure 5).

To identify the optimal design of the device for particle trapping, different geometric parameters were systematically compared with a set of experiments. In particular, the channel length before the first reservoir and the channel aspect ratio were fabricated and tested at different flow rates (from 2 to 11 mL/min), in order to study how these parameters influenced the inertial focusing and vortex flow patterns. They found that as the length before the reservoir was increased, the particles were more accurately focused, closer to the channel wall, increasing their chance to cross the separatrix and enter into the vortices, thus enhancing capture capability and reproducibility. Furthermore, higher aspect ratio AR rectangular micro-channels have been demonstrated to provide better capture efficiency, confirming previous results [41]. For high AR, particles are focused at two positions centered near the long channel walls. Under the right flow conditions, these particles can pass through the separatrix and enter in the recirculating vortex regions. In contrast, for square cross-section channels, the particles are distributed along four focusing positions (top, bottom and both side positions). Consequently, only the lateral particle streams can be trapped by the vortices, while the top and bottom ones flow undisturbed. In addition, the flow rate was also found to affect the capture efficiency, which is maximum for an optimal range. As previously found by [62], the vortices evolution depended on the flow rate, finally occupying the entire reservoir. Increasing the flow rate, the vortex center was progressively shifted towards the back of the reservoir.

Then the capture efficiency decreased, due to the larger particles, which hit the back wall leading to unpredictable trajectories, causing particles to re-enter into the main flow, rather than to move them laterally into the vortex region. Therefore, this design was particularly appropriate for capturing rare cells, and is more suitable for applications where there is the need to capture as many cells as possible. A dependence of the particle trapping efficiency on the reservoir position was also detected. This was probably due to the deformation of PDMS at high pressures, which modified the channel aspect ratio as well as the focusing position [86]. Due to the low elastic modulus of PDMS, at higher flow rates the channels cross-section undergoes deformation, losing the focusing.

Exploiting the size difference between cancer cells and blood cells, the authors demonstrated a high-throughput and label-free capture of cancer cells spiked into healthy human blood, independently of marker expression, suggesting that this approach can be used for the extraction of a large range of tumor cell types. A purity of the extracted sample of 80–100% has been demonstrated, in spite of a lower capture efficiency compared to other techniques, which can be compensated by processing a larger blood volume.

**Figure 6:**
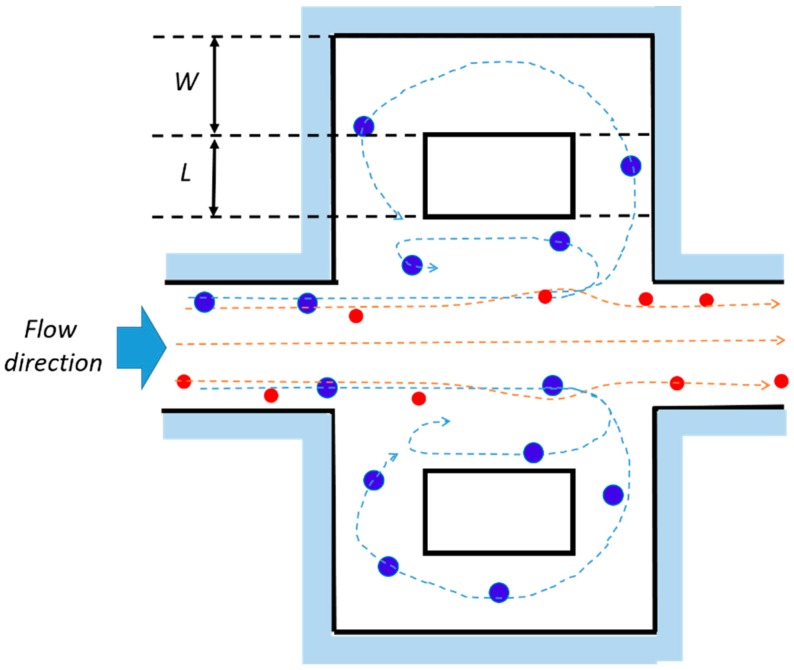
Schematic layout of the reservoir geometry tested by Paiè et al. [80]. Lateral channels of length *L* were added to the standard reservoirs [85] and joined together by a connection channel *W* wide. The dashed paths indicate the fluid streamlines followed by the big (blue) and small (red) particles. Adapted from [87].

New reservoir layouts have also been investigated by Paiè et al. [87], in order to increase the trapping efficiency of the chip in Figure 5. Indeed, the billions of blood cells, in their flow through the device, can still interfere and interact with the target cells trapped in the vortices, thus affecting their trapping stability. In order to limit the particle–particle interactions, side channels were added to the standard reservoirs of [85], as shown in Figure 6.

These channels, by splitting the fluid stream lines, influence the path of the cells trapped in the vortex and force some of them to flow in the lateral fluidic circuit, where they remain trapped over many cycles of the main vortex. Through simulations and experimental validations, the authors have been able to properly choose the lateral fluidic circuit dimensions (namely *L* and *W* in Figure 6), in order to increase the maximum number of particles that can be stably trapped in each reservoir, and consequently, the total efficiency of the chip.

A trapping efficiency 19% higher than the value obtained by the standard Vortex chip [85] has been demonstrated using fluorescent beads. Also in this case, the trapping efficiency was found to be highly dependent on the reservoir position in the chip. This phenomenon was ascribed to the PDMS deformation effects, suggesting that a higher efficiency could potentially be reached with a rigid substrate which prevents from the flow-induced deformation at high flow rates.

**Figure 7:**
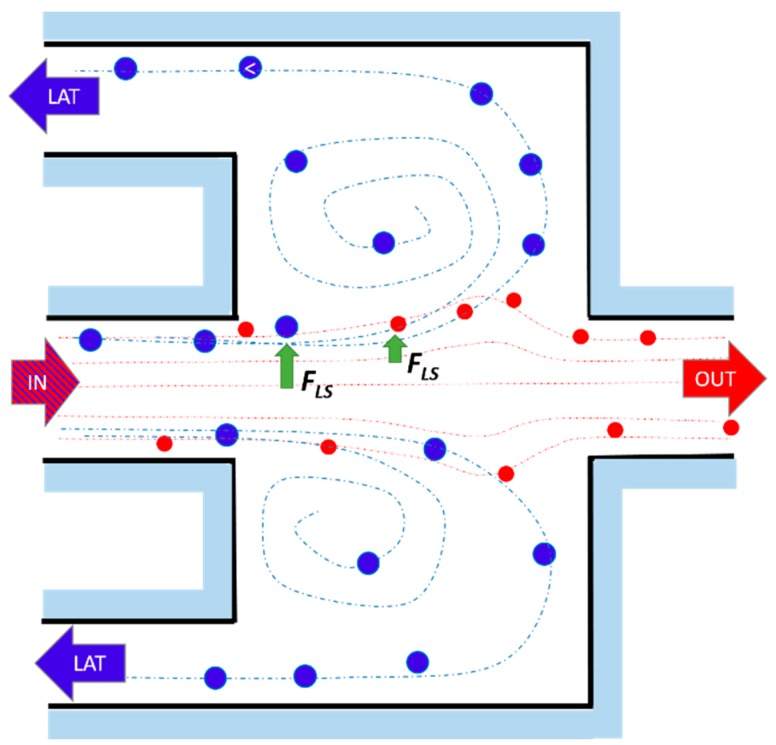
Scheme of the size-selective separation in micro-chambers with three outlets proposed by Wang and Papautsky [88]. At the chamber entrance, the particles are arranged in a two-focus position near the channel walls. The large particles (blue) undergo a larger shear-gradient induced lift force, being pushed in the chamber and exiting through the two later outlets (LAT), while the smaller particles (red) remain in the main channel exiting through the main outlet (OUT). The dashed paths indicate the fluid streamlines followed by the big (blue) and small (red) particles. Adapted from [88].

**Figure 8:**
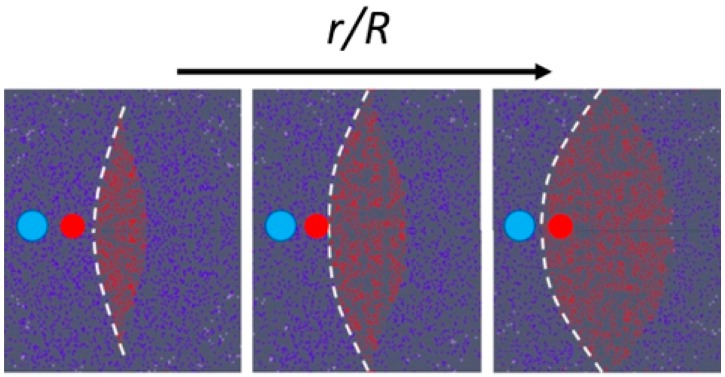
Cross-sectional view of the boundary surface (white dashed line) at the entrance of the micro-chamber increasing the *r*/*R* ratio. The red area represents the main flow exiting through the main outlet, while the blue area indicates the flow exiting through the lateral outlets. Increasing the *r*/*R* ratio, the boundary shifts toward the channel walls, allowing the sorting of particles of different dimensions. Adapted from [89].

A micro-fluidic chip based on inertial migration that achieves an efficient multimodal separation of mixtures of three types of micro-particles with small differences in size was demonstrated by Wang et el. [88] on PDMS. Their approach improved the designs described above, based on a straight micro-channel to pre-focus micro-particles, followed downstream by a channel expansion (micro-chamber), adding lateral siphoning outlets (Figure 7), thus offering a continuous, washing step-free operation device without any vortex saturation effect. Their numerical and experimental results showed that the position of the boundary streamline (i.e., the separatrix) could be precisely varied by tuning the input flow velocity *U_m_* and engineering the channel fluidic resistance ratio *r*/*R*, where *r* is the resistance of a side outlet channels, and *R* is the resistance of the main outlet. Figure 8 shows the position of the separatrix (white dashed line) at increasing values of the *r*/*R* ratio. At a low *r*/*R*, the initial equilibrium position of all particles is within the flow exiting from the lateral outlets, leading to a non-selective extraction of all particles. As the *r*/*R* ratio increases, the boundary streamline moves towards the channel walls, leading to a possible size-based separation. In particular, the authors found that the cutoff diameter *a_c_* increases with *r*/*R*, following the relationship $a_{c}\propto {\left(\frac{r}{R}\right)}^{1/2}$. Furthermore, the experimental results with synthetic micro-particles suggest that *a_c_* scales as the first order of the input flow velocity $a_{c}\propto U_{m}$. This offers the possibility of tuning the cutoff diameter through the input flow velocity and the resistance modification of the outlet channels. These results were also supported by the work of Volpe et al. [89], where 3D computational fluid dynamics (CFD) simulations based on the lattice Boltzmann method were exploited to evaluate the performance of a micro-fluidic device, specifically designed to trap and extract particles by inertial focusing and microscale vortices, and whose scheme is similar to that in Figure 7. The simulations allowed them to characterize the flow properties of the micro-fluidic device as a function of the Reynolds number (Re), the input flow velocity, the chamber dimensions and the outlet channels resistance ratio *r*/*R*. In particular, they found a shift of the separatrix towards to the channels walls when *r*/*R* increases, with a consequent increase of the cutoff diameter, as also confirmed by the experimental results.

**Figure 9:**
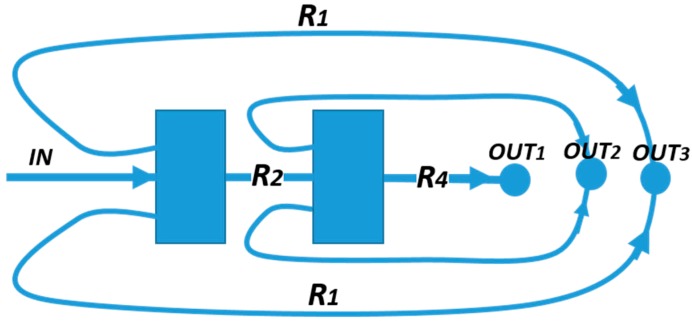
Inertial micro-fluidic multimodal separation designed by Wang et al. [81]. The micro-fluidic resistance network comprises two sorting chambers and three exits for particles, relying on their dimensions. Adapted from [88].

To achieve multimodal separation, in [88] a combination of two (Figure 9) chambers in series has been developed. The resistance ratio was modulated in order to finely tune the cut-off diameter in each chamber. The capability of separating a heterogeneous sample into three distinct distributions was demonstrated by injecting a mixture of micro-particles with diameters continuously distributed in the range from 10 μm to 27 μm.

**Figure 10:**
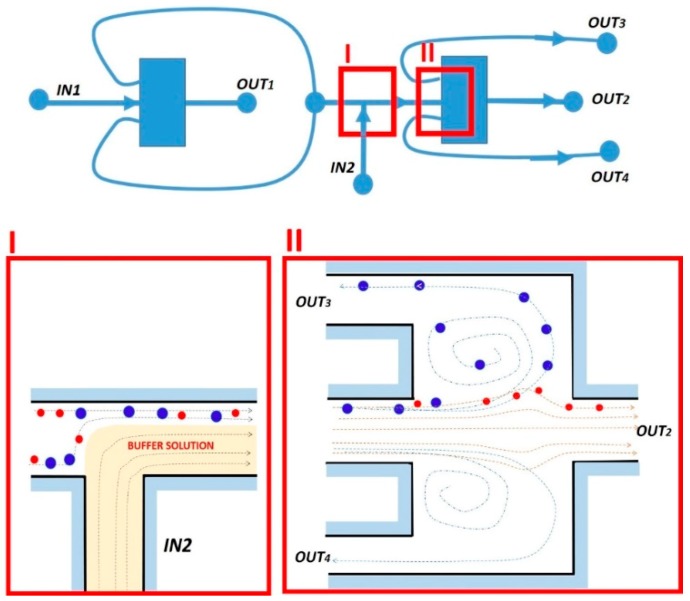
Schematic picture of two vortex sorting units integrated into a device for double sorting and purification. In the insets, a schematic detail of the device operation (I) at the second inlet stage IN2, and (II) in the second chamber. Adapted from [90].

A similar continuous vortex-aided sorting device, integrating two units by connecting the outlets of the first unit into the inlet of the second unit, has also been tested (Figure 10) [90].

The device operation can be summarized as follows: The first sorting unit splits the larger cells from the smaller background cells. To get further purification, the sorting product flows downstream from the side outlets of the first unit into the second unit, where a buffer solution is introduced from inlet 2 to accelerate the flow. By properly designing the fluidic resistance of the network, the condition for sorting the target cells larger than a cutoff diameter are created. After a double-sorting process, the target cells are thus extracted from outlet 3 (O3). The remaining small background cells flow through outlet 2 (O2). The feasibility of rare cells sorting, as well as the efficient removal of a large number of background cells, has been demonstrated for the continuous sorting of spiked human cancer stem-like cells from human blood with >90% efficiency and enhanced purity, as well as for the removal of red blood cells with ~99.97% efficiency [90]. Also in this work, the authors reported a channel deformation during the device operation due to the poor rigidity of PDMS. To this phenomenon could be ascribed the changes in channel resistance strongly affecting the cutoff diameter, which, nevertheless, could be compensated by slightly tuning the flow rate. The main advantage of this vortex-based device compared to the conventional ones was the continuous nature of the device operation, which led to high throughput and high volume applications. Conversely, owing to the presence of the lateral outlet channels, this approach prevents the possibility of packing many reservoirs in a compact footprint that would allow us to parallelize the sorting process and decrease the sample processing time.

**Figure 11:**
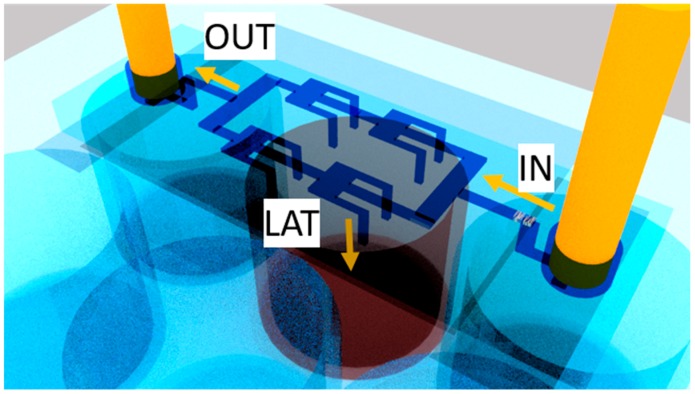
3D sorting Lab on a Chip developed by Volpe et al. [75] on PMMA through fs-laser technology. The particles exiting through the lateral outlets (LAT) are collected in a multi-well plate, while the others flow through the main exit OUT. The ratio between the resistance of the LAT and the OUT is 20. The channels are 60 μm high and 50 μm wide. The chamber’s dimensions are 480 μm × 540 μm. Adapted from [83].

The work of Volpe et al. [83] aimed at improving the throughput of previous implementations, while keeping the sorting efficiency high. In particular, the micro-fluidic devices presented in [83] consisted of micro-channels with expansion chambers in series. Partially inspired from the work of Wang and Papautsky [88], the chambers were supplied with siphoning outlets exiting from the backside of the chip. Relying on the simulation results presented in [89], the dimensions and number of chambers were varied, and different collecting geometries investigated at different flow rates. The layout with maximized trapping efficiency and enhanced throughput (Figure 11) comprised two-branch with two chambers for each of them, one inlet IN, one main outlet OUT and eight lateral outlets LAT.

The channels were 60 µm high and 50 µm wide, meanwhile the chambers had a width of 540 µm and a length of 480 µm. The drops coming out from the lateral outlets were directly collected in the multi-well plate with no tubes, thus increasing the simplicity of the experiments. The device characterizations were performed using separately 6- and 15-µm fluorescent beads, whose dimensions mimic the red blood cells and the CTC ones, respectively. About the 84% of the injected bigger particles were collected by lateral outlets, while just 10% of the smaller ones exited through the main one, thus preserving the purity of the sample.

This quasi-3D design demonstrates the possibility of multiplying the throughput of the device, by simply adding parallel micro-chambers. Furthermore, this LoC is fully inertial, reducing the complexity of other previously-reported, high-throughput devices [91,92]. It is worth mentioning that in the latter work, the fs-laser micro-machining technology was employed to fabricate the device. This technology offers the possibility to choose other polymers than PDMS as substrate material. In this work, PMMA was used, which is a biocompatible, transparent and much stiffer polymer than PDMS, thus withstanding higher flowing pressures without suffering from deformations.

## 3. Conclusions and Perspectives

In this work, a review on the evolution of the inertial particle sorting combined with microscale laminar vortices technology has been presented.

Recent works on micro-fluidic sorting techniques based on the combination of inertial focusing in a straight micro-channel and vortex technology have been critically presented, evidencing the limits and opportunities of each design. As emerged by this excursion, the main drawbacks of the first prototypes, i.e., the limited number of particles trapped per vortex and the deformation of the chamber at high pressure, have been gradually overcome, adding syphoning outlet and changing the fabrication technology from soft lithography to ultrashort laser micro-machining, and consequently, the material from PDMS to much stiffer polymers as PMMA. Furthermore, the possibility for increasing the throughput, without affecting the purity of the sample, has been demonstrated through a parallelization of the extraction process.

Inertial sorting combined with vortex trapping presents several pros in terms of performance, fabrication and operation with respect to other sorting techniques. In fact, it requires neither further mechanical nor electric parts, nor obstacle structures for filtering particles. Moreover, it is label-free, and employs only a simple single-layered micro-fluidic device, offering the possibility to parallelize the sorting process for reaching high purity and throughput. Furthermore, this technique exploits just passive fluid physics, without any complex high-resolution features, instrumentation or surface chemistry.

Thus, the per-sample cost for one chip provided of this pressure control system to run samples is expected to be much lower than ~$500 per test with respect to other traditional systems. Furthermore, this technology can be applied to various samples and cancer types, without adversely damaging the cells and ensuring high-purity. All of these advantages make the combination of inertial focusing in a straight micro-channel and vortex technology a successful technology in a clinical setting. The next step for realizing this goal will be the automation of the device operation, which is especially important for clinical adoption even by the non-specialized user.
